# Professor Frank H. Hamilton

**Published:** 1886-09

**Authors:** 


### EDITORIAL.

FRANK HASTINGS HAMILTON, M. D., LL. D.

Professor Hamilton died at his residence in New York city, on Wednesday, August 11, after a brief illness of two weeks. For the past two years he had suffered from pulmonary phthisis.

He was born in Wilmington, Vt., September 10, 1813. He entered the Medical Department of the University of Pennsylvania, and graduated when only twenty years old. He successfully held professorships in the Fairfield (N. Y.) Medical School, Geneva (N. Y.) Medical College, and the Medical Department University of Buffalo. He was one of the founders of the Bellevue Hospital Medical College, and occupied the chair of surgery in it until 1875.

During his life, and at the time of his death, he held many important positions. In 1881 he was brought prominently before the lay public as one of the consulting surgeons in the case of President Garfield in his last illness.

Professor Hamilton was not only a brilliant surgeon, but a distinguished writer and teacher. His "Treatise on Fractures and Dislocations" is one of the best books on that subject in existence. The third edition of his "Treatise on the Principles and Practice of Surgery" has just been published.

At the last meeting of the Chicago Medical Society, resolutions of respect and condolence were adopted.

